# Time in terms of space

**DOI:** 10.3389/fpsyg.2013.00554

**Published:** 2013-08-26

**Authors:** Asifa Majid, Alice Gaby, Lera Boroditsky

**Affiliations:** ^1^Centre for Language Studies, Radboud University NijmegenNijmegen, Netherlands; ^2^Language and Cognition Department, Max Planck Institute for PsycholinguisticsNijmegen, Netherlands; ^3^School of Languages, Cultures and Linguistics, Monash UniversityClayton, VIC, Australia; ^4^Department of Linguistics, University of California, BerkeleyBerkeley, CA, USA; ^5^Department of Psychology, Stanford UniversityStanford, CA, USA

**Keywords:** time, space, cross-cultural, metaphor, linguistics, linguistic relativity, gesture, bilingualism

Across cultures, people use spatial representations for time: graphs, time-lines, clocks, sundials, hourglasses, calendars, etc. In language, time is also closely tied to space, with spatial terms often used to describe the order and duration of events. In English, we move the meeting forward, push deadlines back, attend a long concert or go on a short break. People make spatial gestures when talking about time, and spontaneously invoke spatial representations when processing temporal language. The papers in this collection shed new light on these time-space mappings in language, gesture, and non-linguistic thought.

The impetus for this collection was the finding by Boroditsky and Gaby (2010) that speakers of Australian languages with absolute spatial reference systems represent time along an east-west axis rather than using a relative spatial axis provided by their bodies. This was surprising because all previous studies in other cultures had found spatial representations of time that were relative to the body. In order to investigate further the possible variability in space-time mappings across cultures, a series of standardized linguistic and nonlinguistic tasks were developed and published in the L&C Field Manuals and Stimulus Materials [fieldmanuals.mpi.nl], which led to the research reported in many of the papers featured in this volume.

Both space and time are complex domains of considerable salience and frequency in conversation. They are almost always grammaticized in languages. The papers in this collection provide new information about how time and space are expressed in little-known languages, and thus provide an important resource for scholars interested in linguistic mappings. So, while English speakers allude to time on a horizontal axis, as shown in the examples above, Mandarin also has a linguistic metaphor which places time on a vertical axis where the past is up and the future down, explored by Lai and Boroditsky (2013), and Bergen and Chan Lau (2012). In Tzeltal, however, a language spoken in the mountains of Mexico, Brown (2012) shows that the future is up(hill) and the past down(hill) (see also Núñez et al., 2013), suggesting that the mapping of time-space on the vertical access may have no natural bias.

Although it has been assumed by many that space-time metaphors, such as these, are universal, the papers by Fedden and Boroditsky (2012), and Gaby (2012), amongst others, show surprisingly few such metaphors. This outcome is tantalizing, raising the question of whether scarcity of time-space mappings in language might also suggest a different way of mapping time to space in thought. Gestural data can be insightful on this point. Where linguistic metaphors of space-time are limited, authors in this volume examined co-speech gesture. Both Levinson and Majid (2013) and Le Guen and Pool Balam (2012) found that outside of literal gestures to the position of celestial bodies in the sky to refer to the time of day, there was little use of gesture space to map out time. This suggests that a focus on absolute frames of reference in spatial cognition and gesture may to some extent pre-empt the use of gesture space for other domains like time.

In order to further explore this issue, many of the papers explicitly tested how speakers organize temporal sequences in space when language was not invoked. Speakers were presented with a set of cards depicting a temporal sequence unfolding and were asked to put them in order. This task requires people to choose a spatial layout for time. Across communities, we see that speakers' spatial layouts conform to the writing direction prominent in the community. So, for example, English speakers display virtually 100% left-to-right ordering, consistent with the communities' orthographic conventions. This confirms earlier studies (e.g., Tversky et al., 1991) but with a much broader sample of cultures than has previously been studied.

Writing conventions vary across communities—not all scripts follow the English left-to-right ordering. Chinese, for instance, has used top-to-bottom, right-to-left, and left-to-write orderings in different places and different points of time. Bergen and Chan Lau (2012), and de Sousa (2012) show that Chinese speakers spatialize time in accordance to the specific exposure to these systems they have had, providing additional evidence that writing direction is an important factor in establishing space-time mappings.

The first four papers in this volume focus primarily on “big” languages, such as English and Chinese, where the speaker populations number the 100 millions. Although considerably different from each other in many aspects, these communities are similar in that widespread literacy is the norm. However, this is not the case in all communities worldwide, and, in fact literacy is a recent innovation for the human species. Studies show that the brains of literates undergo considerable restructuring in comparison to non-literates (Carreiras et al., 2009), raising the question of what sorts of time-space mappings we might see in persons not contaminated by literacy, and with few linguistic time-space mappings.

Later papers in this collection shed some light on this question. They explore “small” languages with speaker numbers in 10 to 100,000 s—an order of magnitude smaller than English or Chinese—where people still live traditional lifestyles as hunter-gatherers or subsistence farmers. It turns out that where writing and reading is not an everyday activity, the time-space mappings within a community show much more variability. Left-to-right is increasingly predominant with increased literacy, but speakers of Kuuk Thaayorre (Australia), Mian (Papua New Guinea), Yélî Dnye (Papua New Guinea), Tzeltal (Mexico), and Yukatek (Mexico) all exhibit myriad other strategies: right-to-left, near-to-far, far-to-near, east-to-west, west-to-east, uphill. In communities that rely on absolute spatial frames of reference in language (Mian, Yélî Dnye, Tzeltal) researchers do find evidence for absolute spatial representations of time as well (a pattern not observed in languages like English or Dutch). However, individual variation is rampant, as is intra-individual variation. This variability showcases the flexibility of time-space mappings, and the large number of potential features of linguistic and extra-linguistic experience that can contribute to how an individual constructs the idea of time in the moment [and Bender et al. (2012) show the complexity of establishing these mappings].
